# Elastomer-Embedded Multiplexed Optical Fiber Sensor System for Multiplane Shape Reconstruction

**DOI:** 10.3390/s23020994

**Published:** 2023-01-15

**Authors:** Arnaldo Leal-Junior, Leandro Macedo, Leticia Avellar, Anselmo Frizera

**Affiliations:** Graduate Program in Electrical Engineering, Federal University of Espírito Santo, Vitória 29075-910, Brazil

**Keywords:** polymer optical fibers, shape reconstruction, optical fiber sensors

## Abstract

This paper presents the development and application of a multiplexed intensity variation-based sensor system for multiplane shape reconstruction. The sensor is based on a polymer optical fiber (POF) with sequential lateral sections coupled with a flexible light-emitting diode (LED) belt. The optical source modulation enables the development of 30 independent sensors using one photodetector, where the sensor system is embedded in polydimethylsiloxane (PDMS) resin in two configurations. Configuration 1 is a continuous PDMS layer applied in the interface between the flexible LED belt and the POF, whereas Configuration 2 comprises a 20 mm length PDMS layer only on each lateral section and LED region. The finite element method (FEM) is employed for the strain distribution evaluation in different conditions, including the strain distribution on the sensor system subjected to momentums in roll, pitch and yaw conditions. The experimental results of pressure application at 30 regions for each configuration indicated a higher sensitivity of Configuration 1 (83.58 a.u./kPa) when compared with Configuration 2 (40.06 a.u./kPa). However, Configuration 2 presented the smallest cross-sensitivity between sequential sensors (0.94 a.u./kPa against 45.5 a.u./kPa of Configuration 1). Then, the possibility of real-time loading condition monitoring and shape reconstruction is evaluated using Configuration 1 subjected to momentums in roll, pitch and yaw, as well as mechanical waves applied on the sensor structure. The strain distribution on the sensor presented the same pattern as the one obtained in the simulations, and the real-time response of each sensor was obtained for each case. In addition, the possibility of real-time loading condition estimation is analyzed using the k-means algorithm (an unsupervised machine learning approach) for the clusterization of data regarding the loading condition. The comparison between the predicted results and the real ones shows a 90.55% success rate. Thus, the proposed sensor device is a feasible alternative for integrated sensing in movement analysis, structural health monitoring submitted to dynamic loading and robotics for the assessment of the robot structure.

## 1. Introduction

While electronic sensors have advanced in recent years, including flexible electronics on different substrates [1], optical fiber sensors remain an increasingly relevant approach in a wide range of applications, from environmental monitoring to medical devices [2]. Due to the small dimensions and weight of optical fibers, as well as their electromagnetic and galvanic isolation, these sensors have become widely used in the integration of different materials [3]. Moreover, such advantages also enable their use in wearable systems and healthcare systems [4]. A further advantage of their electromagnetic field immunity is the possibility of using such sensors in electric motor assessment [5] and in conjunction with assistive robots (which are generally equipped with electric actuators), as well as with devices that emit electromagnetic waves [4].

The majority of sensor systems are applied in silica optical fibers, which present low optical loss, but they are brittle and have low impact resistance and strain limits [6]. By utilizing polymer optical fiber technology (POF), these drawbacks can be mitigated, since different materials of POFs can be developed which have higher strain limits, flexible features and impact toughness due to advances in polymer processing, preparation and fabrication [7]. Its rough surface characteristics are responsible for its easy incorporation into textiles and resins, as shown in many reports on wearable sensors for human health assessment [8,9,10]. Furthermore, POFs have a lower Young’s modulus than silica fibers (leading to enhanced flexibility), which can be further increased when POFs are fabricated from highly flexible materials [11].

There have been many different approaches to sensing applications with POFs over the years, which closes the gap between the silica optical fibers and POFs regarding the possibilities of sensor applications approaches. Such approaches include interferometers [12], long-period gratings [13], fiber Bragg gratings (FBGs) [14], evanescent waves [15], intensity variation [16] and nonlinear effects [17].

For the distributed optical fiber sensors, the generally applied techniques include optical time-domain reflectometry (OTDR) and optical frequency-domain reflectometry (OFDR) [18]. It should be noted that such techniques have issues related to their spatial resolution of a few meters, which can be increased by using nonlinear effects or optical backscattered reflectometry, but such techniques need bulky and expensive hardware [19]. Furthermore, OFDR generally requires bulky hardware, such as swept-laser interferometers and microwave photonic circuits [20]. In another approach, quasi-distributed systems are obtained with multiplexed point-care sensors such as FBG arrays, which are capable of high-resolution sensing due to their multiplexing capabilities in conjunction with their high resolution [21].

An important and ever increasing sensing application is the use of shape reconstruction sensors with decisive advantages in structural health monitoring [22], biomechanical applications [23], biomedical applications (even in needle shape reconstruction [19]) and even in the assessment of continuous robot positions [24]. However, the current methods for shape reconstruction applications involve the use of strain gauges distributed along the structure, which have important drawbacks due to complex assembly (including the electric wires and gluing methods) and difficulties in the signal processing related to the lack of multiplexing capabilities of such sensors [25]. Considering the aforementioned advantages of optical fiber sensors, the use of such sensor approaches for shape reconstruction applications were proposed in the literature, mainly using FBGs [22] and their variants based on radial positioning [26], as well as sensor distribution along the monitored structures for 2D/3D shape reconstruction [27]. Furthermore, tilted FBGs [28] and cladding-mode-based [29] approaches were presented for shape reconstruction analysis, whereas the use of optical backscattering reflectometry is also applied with millimeter-range spatial resolution [19]. However, despite the necessity of specialized equipment for grating inscription, the interrogation equipment is bulky and generally nonportable [30].

To address these issues, a cost-effective distributed optical fiber sensor was proposed and analyzed using experimental and analytical methods in [31], where a flexible lamp belt with light-emitting diodes (LEDs) is side-coupled to a POF with lateral sections with sequential activation of the LEDs for the decoupling of sensor responses. In this case, the curvatures in the lateral section of an optical fiber in the regions at which there is a side-coupled LED can be estimated in a multiplexed approach, where the analytical models for optical power variation for curvature sensing using POFs are described in [32,33]. The photodetectors are connected to each end facet of the fiber, and the forward and backward optical power are measured and compared when each LED is activated. This technique enables the development of a quasi-distributed sensor system with self-reference (reducing errors caused by light source deviation), and this multiplexing approach was previously employed to develop a portable instrumented insole for assessing plantar pressure and ground reaction force [34], as well as smart textiles [35] and smart carpets [36].

This paper presents the development of an optical fiber sensor system for shape reconstruction using a multiplexed intensity variation based approach. The sensor system comprises 30 sensors distributed in a flexible light-emitting diode (LED) belt embedded in a polydimethylsiloxane (PDMS) resin. A comparison between the system with a continuous PDMS layer and the other with a non-continuous layer is presented as a function of the applied force on each sensor. Then, different loadings are applied in the sensor system, which is able to distinguish the loadings and classify the type of mechanical loading in the POF sensors. In addition, the strain transmission along the optical-fiber-embedded sensor system is obtained using a low-cost approach with the possibility of shape reconstruction with spatial resolution of a few millimeters. Thus, the proposed approach includes a novel method for shape reconstruction using polymer optical fibers, which present intrinsic advantages of portability and lower cost. In addition, the use of machine learning methods (such as the unsupervised method for clusterization) indicates another contribution on shape reconstruction applications using the optical fiber sensors.

This paper is organized as follows. After the introduction, the materials, method for sensor fabrication and the numerical and experimental setup are presented in Section 2. Then, the numerical and experimental results and their discussions are depicted in Section 3. Finally, final remarks and future works are presented in Section 4.

## 2. Materials and Methods

The proposed sensor system comprises a polymethyl methacrylate (PMMA) POF (980 μm core diameter and 10 μm cladding thickness) with 30 lateral sections to expose its core to the side coupling with the LED from a flexible lamp belt. One end of the POF is connected to a phototransistor IF-D92 (Industrial Fiber Optics, Tempe, AZ, USA). The FRDM-KL25Z (Freescale, Austin, TX, USA) microcontroller is responsible for the LED’s sequential activation as well as performs the signal acquisition, where the signal acquisition frequency is 15 Hz and the LED’s activation frequency is 10 Hz. The signal acquisition is performed when each LED is active. Such an approach enables an independent response for each sensor, as demonstrated and validated in [31]. In addition, the use of two photodetectors, one at each end facet of the POF, leads to the possibility of a relative compensation for the LED fluctuation, since the data are obtained from the difference between both photodetectors.

**Figure 1:**
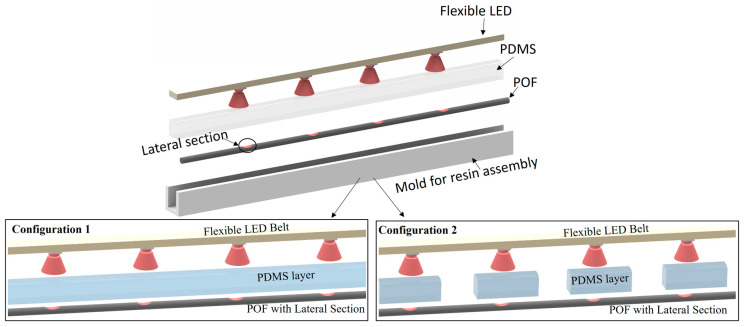
Sensor assembly for each configuration.

For the sensor assembly, the sequential lateral sections are performed in the POF through the abrasive removal of material following a controlled lateral section length and depth. In addition, a flexible LED belt is placed close to the optical fiber with its LEDs aligned with the lateral sections of the POF, as presented in Figure 1. The flexible lamp belt and the POF are positioned in an enclosed mold, where a PDMS precursor is added by the combination of the monomer and curing agent in a 10:1 proportion. The resin is cured after 24 h and the sensor system is removed from the mold, resulting in the coupling between the optical fiber and the flexible LED using a transparent medium.

The physical principles for the sensor operation are based on the optical property variation on the POF when subjected to pressure, force and/or curvature. In this case, the displacement or strain, especially curvatures in the optical fiber (caused by different mechanical loadings), lead to variations in the critical angle (considering the analysis using geometric optics) as well as the optical path, which lead to variations in the transmitted optical power (as depicted and modeled in [32]). Moreover, the stress-optic effect also occurs when the optical fiber is under mechanical stress, leading to a variation in the refractive index of the fiber, which can also lead to variations in the optical responses due to the mechanical loadings [32].

To study the mechanical behavior of the sensor, the finite element method (FEM) was used in the software Ansys 2019 R3. A first analysis was carried out by simulating a beam in a clamped-clamped condition with a load applied to half of the beam length. This analysis was used to investigate the cross-talk effect on the sensor. A second analysis was carried out in a clamped-free condition for the beam. In this case, three scenarios were exploited: roll (moment in x direction applied at the free end of the beam), pitch (moment in y direction applied at the free end of the beam) and yaw (moment in z direction applied at the free end of the beam). For each case, the displacement and the equivalent von Mises strain were calculated by the software.

**Figure 2:**
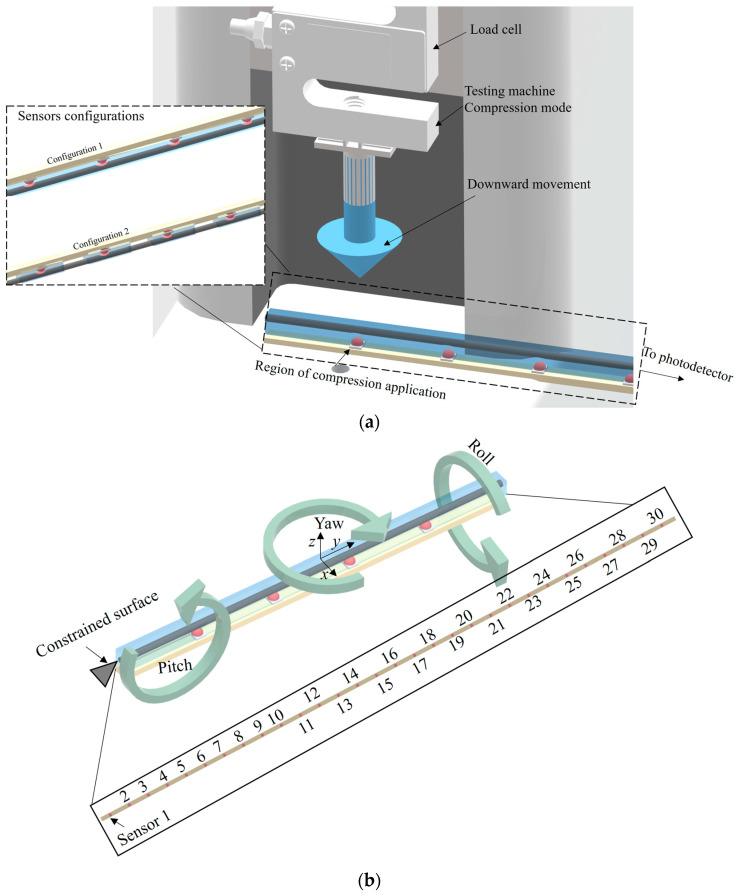
(**a**) Experimental setup for sensor pressure characterization. (**b**) Direction of momentum applied on the sensor system. Figure also shows the sensor positions.

For the experimental evaluation, the sensor systems, using both configurations, were positioned on the universal testing machine at the compression configuration depicted in Figure 2a. Forces from 0 to 120 N in steps of 20 N were applied on each sensor of Configurations 1 and 2. The forces were applied with a support of 25 mm diameter (see Figure 2), which resulted in a pressure of 0 to 243.70 kPa with 48.74 kPa steps. The response of the 30 sensors in Configurations 1 and 2 were compared with respect to the determination coefficient (R^2^) with a linear regression and the sensitivity of each sensor as a function of the applied pressure. As an important parameter in the comparison of different configurations, the cross-sensitivity between sequential sensors (e.g., between sensors 17, 18 and 19) was obtained for each configuration for the maximum pressure tested (244.46 kPa).

Then, mechanical loading/displacements were applied in different axes to demonstrate the sensor system using the Configuration 1 (with continuous layer of PDMS) capability of detecting the strain distribution in different planes, as presented in the arrows of Figure 2b. Furthermore, harmonic loading is applied on the sensor system by means of mechanical wave propagation in the sensor. In these cases, the strain distribution in the optical fiber is obtained and presented for each loading condition.

For the shape reconstruction considering the responses of each sensor in the system, the first step is the normalization to reduce the offset between sensors due to the light coupling between each LED and its respective lateral section. Thereafter, the sensors’ responses are normalized as a function of their sensitivities. The strain distribution at each time was obtained, where it is possible to observe the variation in the strain distribution along the optical fiber sensor system. The datasets for the shape reconstruction algorithm as well as data clusterization were experimentally obtained from the different loadings applied on the sensor system.

It is also worth noting that the responses of the 30 sensors can be used on the assessment or classification of the mechanical loading on the sensor system. Such classification can be achieved using the k-means algorithm using an unsupervised approach, which resulted in the classification through the attribution of indices based on the momentums around each plane. The index 0 is attributed when there is no momentum applied on the axis, whereas 1 is related to the momentum pitch (see Figure 2). Moreover, the indices 2 and 3 are related to the momentums yaw and roll, respectively. In this case, the k-means algorithm is based on the data clusterization in K centroids (used as the prototype of the cluster), where the number of centroids in this analysis is 4, i.e., K = 4. To that extent, each point/observation is attributed to the cluster with smallest mean when compared with all centroids, which results in data partitioning into different Voroni cells (considering the value of k) [37].

## 3. Results and Discussions

**Figure 3:**
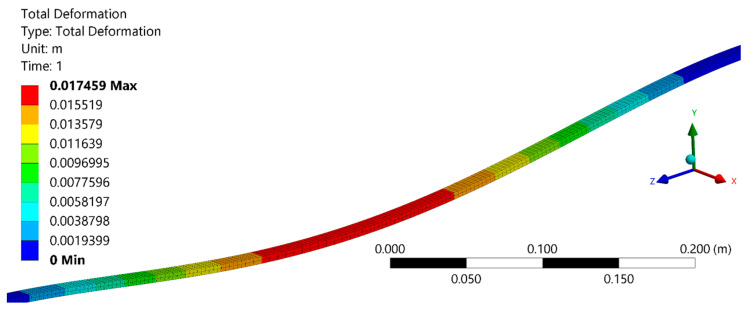
Numerical simulation of Configuration 1 with a force applied on the center of the optical-fiber-embedded sensor system.

As the first analysis of the sensor system, Configuration 1 (using the continuous layer of PDMS) was subjected to a force applied in the middle of the LED strip. Figure 3 presents the FEM results of the numerical simulation using the force applied in the sensor system. The results show that the strain is not concentrated at the center of the sensor system. Actually, there is a strain distribution along the optical fiber. Thus, it is possible to expect that the force applied is distributed to the adjacent sensors following a beam model [38], where there is a displacement distributed along the fiber with a maximum value at the load application region.

**Figure 4:**
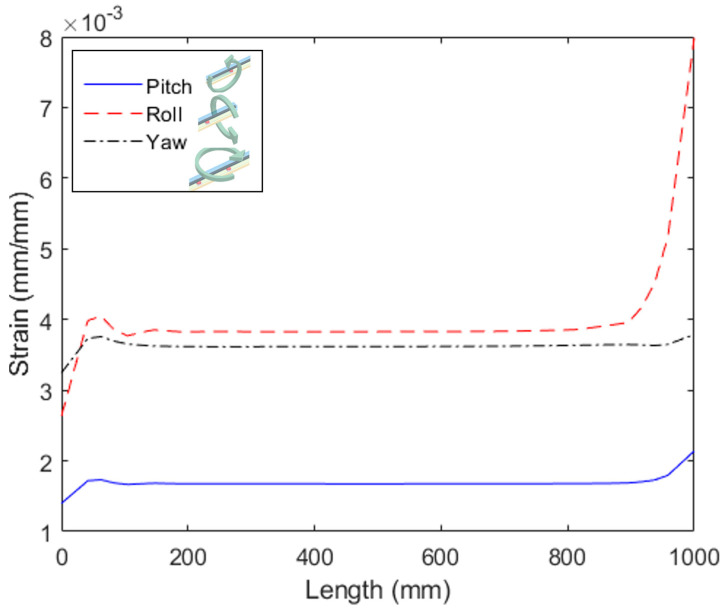
Numerical simulation of Configuration 1 subjected to momentums around each axis.

Similarly, the strain distributions in the sensor system due to momentums applied around reach axis are presented in Figure 4. In these cases, it is possible to observe differences in the strain considering each mechanical loading condition. For the momentum applied around the *x*-axis, the maximum strain obtained is 8.0 × 10^−3^ mm/mm, which is obtained in the region around 900 mm, i.e., close to Sensors 25 to 30 if the positions presented in Figure 2b are considered. In addition, the maximum strain of 1.7 × 10^−3^ mm/mm is obtained in the region close to Sensors 28 to 30 for the momentum applied around the *y*-axis. Finally, the maximum strain obtained for the momentum around the *z*-axis is close to 3.8 × 10^−3^ mm/mm in the region of Sensor 3. Therefore, it is possible to estimate not only the region at which a force (or any mechanical loading) is applied, but also the classification of around which axis the momentum is applied through the strain distribution and values in the 30 sensors.

**Figure 5:**
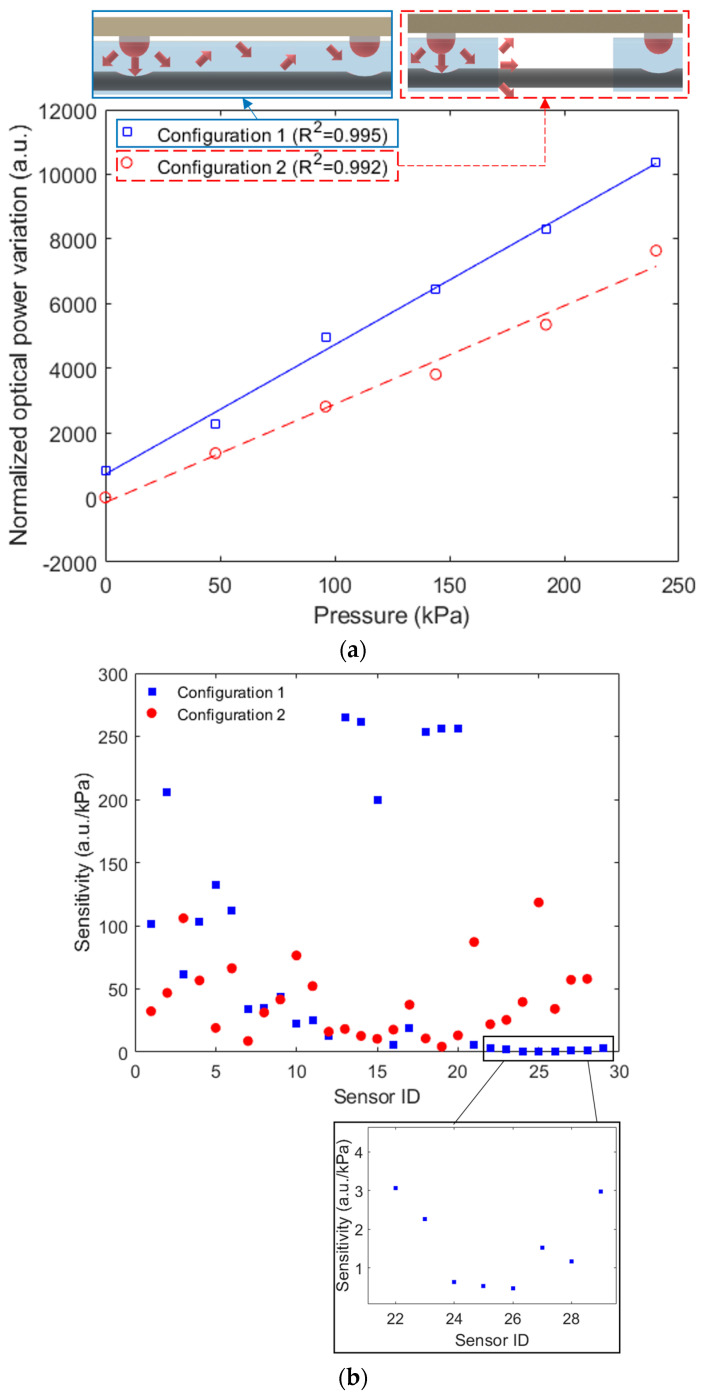
(**a**) Transmitted optical power variation as a function of force for each configuration. Figure inset shows the schematic representation of the light coupling between the adjacent sensors. (**b**) Sensor pressure sensitivities as a function of each sensor ID. Figure inset shows a magnified view of Sensors 22 to 29 in Configuration 1.

The numerical simulations indicate that the cross-sensitivity between adjacent sensors is also related to the strain distribution when a force is applied, leading to small strain in the regions closer to the point at which the force is applied (i.e., the adjacent sensor). Since the force is applied in the continuous region of the LED belt, similar behavior is obtained in both Configurations 1 and 2. For this reason, a cross-sensitivity is expected in both configurations, since the adjacent sensors are close (10 mm distance) in both configurations. In both cases, there is a small thickness of the PDMS layer between the LED and the POF’s lateral section, where the PDMS has the transparency properties to enable the light transmission from the LED to the POF. However, there is an additional feature in Configuration 1 in which the continuous PDMS layer is applied. In this case, the continuous PDMS transparent layer results in the light transmission to adjacent sensors, i.e., if Sensor 13 is activated (see Figure 5 inset), the optical signal of the LED related to Sensor 13 can be transmitted through the continuous PDMS layer and couple to the lateral section of Sensor 14. For this reason, it is expected that Configuration 1 has a higher cross-sensitivity when the responses of adjacent sensors are considered. Considering the 7.5% reduction in the optical power per millimeter, there is a significant optical power coupled to the next two sensors.

In order to experimentally analyze the sensors’ responses as a function of the applied pressure, Figure 5a presents the transmitted optical power as a function of the applied force for Sensor 9 at each configuration. Considering the results in Figure 5a, Configuration 1 presents a higher determination coefficient (R^2^ = 0.995) than the other configuration (R^2^ = 0.992). In Figure 5b, the sensitivities of all sensors are presented for Configurations 1 and 2. In this case, Sensor 13 presented the highest sensitivity of the those of Configuration 1, whereas the highest sensitivity of Configuration 2 was found in Sensor 25. The differences in the sensitivities are related to the POF’s lateral section positioning with respect to its LED, as well as the thickness of the PDMS layer between the pressure section and the LED. In general, the average force sensitivity of Configuration 1 is around 83.58 ± 99.00 a.u./kPa, which is higher than the one of Configuration 2, 40.06 ± 29.76 a.u./kPa.

**Figure 6:**
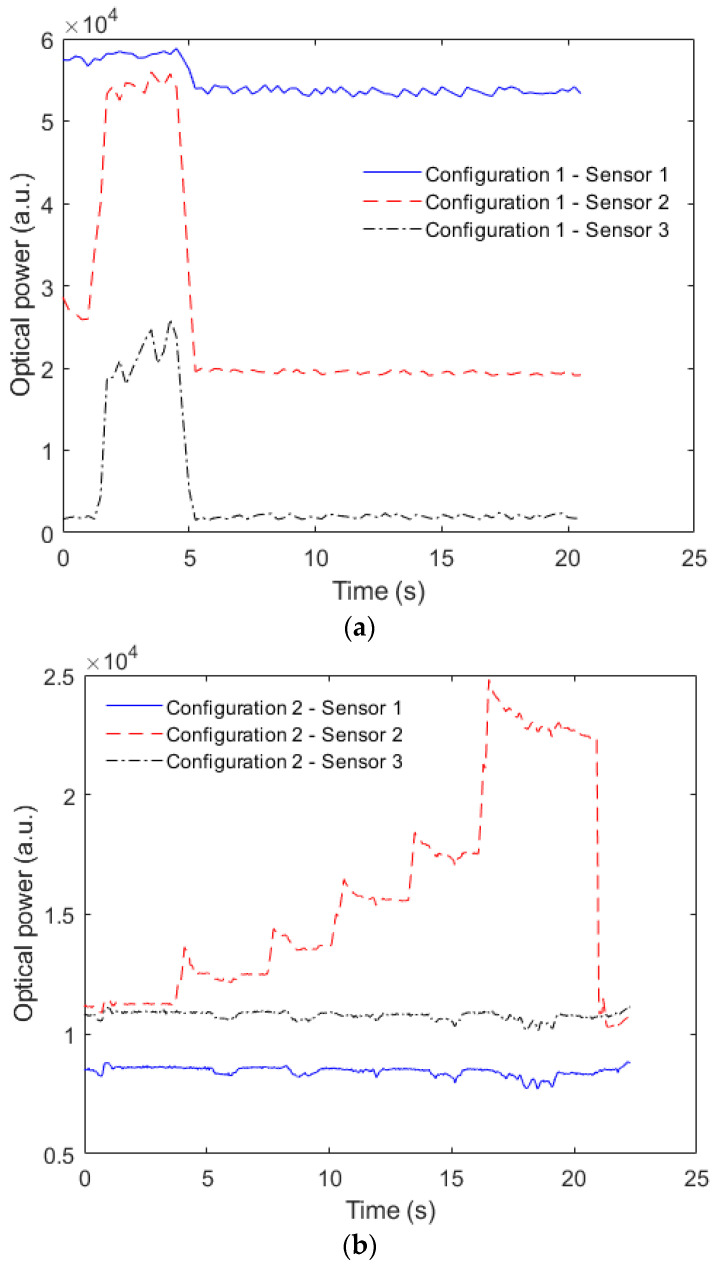
Optical power transmission as a function of time for sensors 1, 2 and 3 considering (**a**) Configuration 1 and (**b**) Configuration 2.

An experimental analysis of the cross-talk between adjacent sensors is presented in Figure 6a for Configuration 1 and Figure 6b related to Configuration 2, where the pressure was applied in Sensor 2 in both cases. The results show a higher cross-talk in Configuration 1, where a 45.5 a.u./kPa cross-sensitivity is obtained. Such cross-sensitivity is higher than the one presented in Figure 6b for Configuration 2 (0.94 a.u./kPa). This higher cross-sensitivity is related to the optical signal coupling to the adjacent lateral section due to the continuous PDMS layer, as anticipated in the Figure 5 inset. However, since there is a strain transmission in both cases when a force is directly applied to the LED belt (coupled to the POF), the cross-sensitivity occurs in both configurations.

**Figure 7:**
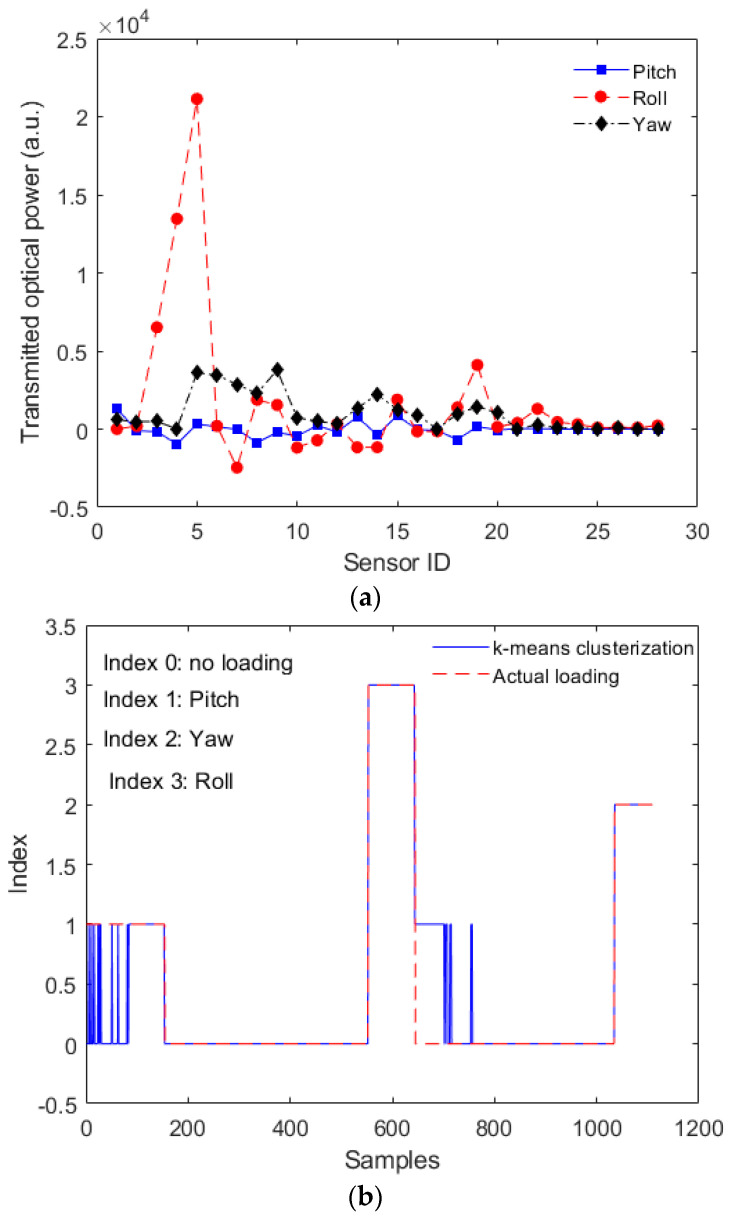
(**a**) Transmitted optical power distribution along the sensor system (considering all 30 sensors) for different loading conditions. (**b**) Clustering results of the sensor responses using k-means. Figure also shows the actual mechanical loading applied for each region.

The smaller cross-sensitivity obtained in Configuration 2 enables its application in localized force/pressure assessment such as the ones in instrumented insoles [34] and multiparameter sensing, in which each sensor measures a different parameter [31]. However, Configuration 1 can be suitable for shape reconstruction applications, where the continuous variations in the adjacent sensor responses due to mechanical and optical coupling enable the reconstruction of the fiber shape considering all 30 sensors. To that extent, the tests applying momentums around different planes were performed in Configuration 1. The responses of all 30 sensors were normalized (considering the sensitivity and initial value), and the sensors responses for each loading condition are presented in Figure 7. In Figure 7a, the strain distribution along the optical fiber is presented for each condition, where it is possible to observe the differences in the strain distribution as a function of each loading condition. In this case, there are similarities on the transmitted optical power variation when compared with the simulation of Figure 4. The major difference in the simulation and experimental results is the roll position, where such difference is due to the point at which the roll is applied, since it is opposite to the one presented in the simulation results (see Figure 4). In addition, the unsupervised approach, using k-means algorithm, enables the clustering of the data in which it is possible to infer the mechanical loading conditions on the fiber. As discussed in Section 2, the indices 0, 1, 2 and 3 represent the no load as well as momentums in the *x*-, *y*- and *z*-axis, respectively. Comparing the clustering in Figure 7b with the actual mechanical loading also presented in Figure 7b, it is possible to confirm that the proposed algorithm correctly classified the data in 90.55% of the cases, where the most common error in the clustering is index 0 classified as index 1, which is related to the similarity on the strain distribution in both conditions, as can be verified in Figure 7a.

**Figure 8:**
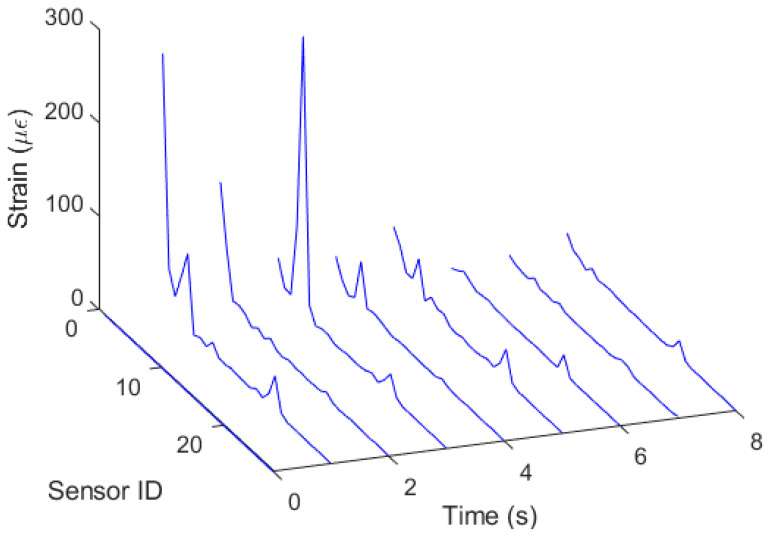
Strain distribution along the optical-fiber-embedded sensor system as a function of time and sensor position.

Then, the sensor system is subjected to periodic strain along the optical fiber by means of a mechanical loading perturbation. In this case, the responses of each sensor were considered to obtain the shape reconstruction of the sensor system subjected to the mechanical wave propagation. Figure 8 shows the distribution in the fiber as a function of time. The results show the shape reconstruction of the fiber in all tests, where there is a sequential increase in the sensor strain with a potential spatial resolution of 3 cm. Such results not only indicate the feasibility of the proposed sensor system on shape reconstruction applications, but also show the possibility of using the system’s low cost and potential scalability of shape reconstruction in complex structures such as robots and in optical-fiber-embedded clothing accessories for movement analysis.

Compared with previously proposed techniques, the multiplexed POF intensity variation-based shape reconstruction sensor system provides intrinsic advantages related to their lower cost (since only inexpensive LEDs, photodetectors and microcontrollers are needed, instead of optical spectrum analyzers) and portability due to the possibility of embedding the detectors in the sensor structure or even in the measured region, since only compact and portable devices are used. In addition, the use of POFs enable technical advantages due to the smaller Young’s modulus (when compared with silica optical fiber) that leads to higher sensitivity, as well as their higher strain limits and non-brittle nature. The flexible construction enables the direct integration of the proposed device in different structures ranging from metallic, concrete, plastics and even textiles. It is also worth mentioning that the sensor system has an easy fabrication using inexpensive devices, equipment and materials. The spatial resolution of the proposed sensor system is in the order of a few centimeters, which is similar to the ones obtained in FBG arrays with the advantage of easier fabrication. Considering the distributed optical fiber sensing, the proposed approach has a spatial resolution close to the one presented in [19] (which presented a spatial resolution of a few millimeters).

## 4. Conclusions

This paper presented the development of a multiplexed POF intensity variation-based sensor embedded in an elastomer for shape reconstruction applications. The sensor system was numerically analyzed using the FEM analysis, where the strain distribution along the sensor assembly in different conditions was characterized. In addition, two different configurations were tested: in the first configuration a continuous PDMS layer was applied in the interface between the flexible LED belt and the POF (with lateral sections), whereas the second configuration comprises a PDMS layer of around 20 mm to cover only the LED and lateral section regions. The comparison between both approaches indicated higher sensitivity of Configuration 1 (83.58 a.u./kPa), whereas Configuration 2 presented the smallest cross-sensitivity between adjacent sensors (0.94 a.u./kPa). Considering such high sensitivity, Configuration 1 shows its suitability for shape reconstruction applications, where momentums considering roll, pitch and yaw are applied in the sensor system, which presented similar responses of strain distribution as the ones predicted by the FEM simulations. In addition, a k-means unsupervised approach was applied as a method for loading condition detection in the sensor system for practical applications. In this case, there is a 90.55% success rate in the correct clustering of roll, pitch, yaw and no loading on the sensor. Another validation was performed by applying mechanical waves on the PDMS-embedded sensor system, which shows its capability of real-time shape reconstruction. Thus, the proposed sensor device is a feasible alternative for integrated sensing in movement analysis, structural health monitoring submitted to dynamic loading and even in robotics for the assessment of the robot structure, including the continuous robots. Future works will include the combined use of additional PDMS-embedded sensor systems for shape reconstruction in smart textiles.

## Data Availability

The data presented in this study are available on request from the corresponding author.
